# Top-down and bottom-up modulation of pain-induced oscillations

**DOI:** 10.3389/fnhum.2015.00375

**Published:** 2015-07-02

**Authors:** Michael Hauck, Claudia Domnick, Jürgen Lorenz, Christian Gerloff, Andreas K. Engel

**Affiliations:** ^1^Department of Neurophysiology and Pathophysiology, University Medical Center Hamburg-EppendorfHamburg, Germany; ^2^Department of Neurology, University Medical Center Hamburg-EppendorfHamburg, Germany; ^3^Faculty of Life Science, Laboratory of Human Biology and Physiology, Applied Science UniversityHamburg, Germany

**Keywords:** pain, EEG, gamma band, attention, functional imaging

## Abstract

Attention is an important factor that is able to strongly modulate the experience of pain. In order to differentiate cortical mechanisms underlying subject-driven (i.e., top-down) and stimulus-driven (bottom-up) modes of attentional pain modulation, we recorded electric brain activity in healthy volunteers during painful laser stimulation while spatial attention and stimulus intensity were systematically varied. The subjects’ task was to evaluate the pain intensity at the attended finger, while ignoring laser stimuli delivered to the other finger. Top-down (attention) and bottom up (intensity) influences differed in their effects on oscillatory response components. Attention towards pain induced a decrease in alpha and an increase in gamma band power, localized in the insula. Pain intensity modulated delta, alpha, beta and gamma band power. Source localization revealed stimulus driven modulation in the cingulate gyrus (CG) and somatosensory areas for gamma power changes. Our results indicate that bottom-up and top-down modes of processing exert different effects on pain-induced slow and fast oscillatory activities. Future studies may examine pain-induced oscillations using this paradigm to test for altered attentional pain control in patients with chronic pain.

## Introduction

Pain is an aversive experience, which inherently attracts attention, disrupting all ongoing activities and thoughts (Eccleston and Crombez, 1999). This feature of pain experience is of evolutionary importance because an immediate action is required if a threat of bodily harm exists. Functional imaging experiments have revealed that attentional brain networks consisting of frontal, parietal and thalamic structures are also engaged during pain processing (Peyron et al., 1999). Moreover, the attention directed to or away from pain seems to have a modulatory effect on activity in somatosensory regions (Seminowicz et al., 2004; Schoedel et al., 2008) and on the subjective perception of painful stimulation (Miron et al., 1989). In particular, the anterior cingulate cortex (ACC) and the insula have been found to be activated during attention to pain or expectation of painful stimulation (Peyron et al., 1999; Sawamoto et al., 2000). The ACC is associated with pain affect (Rainville et al., 1997) and subjective pain intensity and unpleasantness (Coghill et al., 1999; Sawamoto et al., 2000), while the insula and the secondary somatosensory cortex (SII) play an important role in the integration of pain for feelings and behavior (Craig, 2002). Especially pain-induced oscillations in the gamma band (>30 Hz), localized in the insula/SII are modulated by directed attention (Hauck et al., 2007). The reciprocal relationship of pain and attention is of high clinical relevance, as attentional processes seem to be altered in chronic pain patients leading to a preoccupation with their painful symptoms (de Tommaso et al., 2003; Gracely et al., 2004). The ability to actively cope with pain is generally believed to depend on adaptive processing strategies. The concept of hypervigilance assumes an abnormal allocation of attentional resources to pain. This leads to catastrophizing and a failure to disengage attention from pain thereby promoting increased pain intensity as an indication of maladaptive coping (Crombez et al., 2004).

Recent studies indicate that the neuronal mechanism of attention, which leads to the preferred processing of the attended input, relates to synchrony within local assemblies of neurons and across different cortical areas (Singer, 1999; Engel et al., 2001; Salinas and Sejnowski, 2001). The key hypothesis is that phase coherence of neuronal oscillations renders neural communication more efficient and, thus, has a strong impact on signal flow through cortical networks (Fries, 2005). Synchrony has been found to be modulated by bottom-up factors (i.e., stimulus-driven factors like physical saliency or novelty) and by top-down influences (i.e., subject-driven factors like task relevance involving selective attention; Herrmann et al., 2004). In particular, the synchronization of oscillations in the gamma band seems to have a strong impact on target neurons because their high frequency matches optimally the integration time window of cortical neurons (Engel et al., 2001; Jensen et al., 2007). Strong positive correlations between gamma-activity and physical stimulus strength as well as perceived pain intensity have been shown (Croft et al., 2002; De Pascalis et al., 2004; De Pascalis and Cacace, 2005; Gross et al., 2007; Tiemann et al., 2012). Our group recently studied the effects of spatial attention on oscillations induced by intracutaneous electrical stimuli (Hauck et al., 2007) and found enhanced gamma activity and coupling between bilateral somatosensory cortical sites for attended stimuli as measured by magnetoencephalography (MEG). Recent studies in the visual and somatosensory system (Bauer et al., 2014; van Ede et al., 2014) were able to demonstrate differences in frequency responses between attentional predictability and poststimulus attentional enhancement. While prestimulus alpha modulation reflected the predictability of an imminent stimulus, poststimulus gamma modulation seems to be stimulus bound.

In the present study, we used painful laser stimuli and focused the analysis on pain-induced brain oscillations in the electroencephalogram (EEG). In contrast to MEG, which is most sensitive to the tangential orientation of neural dipoles, EEG captures activity from both tangential and radial sources and thus, contains additional information (Bromm and Lorenz, 1998). We systematically varied spatial attention and stimulus intensity. Subjects had to attend to one of the stimulated fingers and to evaluate the intensity of the laser pulses while ignoring a series of equiprobable stimuli at the other finger. We expected both top-down (spatial attention) and bottom-up (stimulus intensity) factors to have an impact on neuronal synchronization in cortical sites engaged in pain processing. The emerging time-frequency response components were localized using linear beamforming (Van Veen et al., 1997).

## Materials and Methods

### Participants

Twenty-one healthy participants (10 female), aged 20–29 years (mean 24.17), participated in this study and received monetary compensation. Basic neurological investigation did not reveal any abnormalities. Subjects were informed that they could terminate the experiment at any time and written informed consent was obtained. The study was conducted in accordance with the Declaration of Helsinki and was approved by the local ethics review board.

### Pain Stimulus, Procedure and Pain Rating

**Figure 1:**
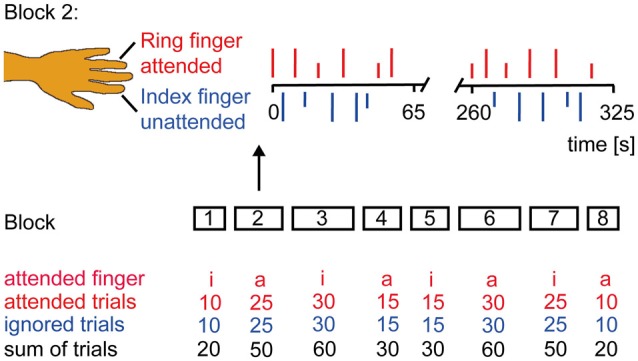
**Experimental Design.** Subjects were instructed to classify the stimulus intensity if the attended finger was stimulated and to ignore the other finger. Subjects’ report via button press was prompted by an acoustic signal 3 s after each laser stimulus. The experiment had a counterbalanced block design. Laser stimuli were delivered in eight blocks of different duration to the left index (i) and ring (a) finger. Block 2 is illustrated in more detail. Occurrence of low (1.5 × pain threshold = short vertical lines) and high (2 × pain threshold = long vertical lines) intensity stimuli was randomized as were the inter-stimulus interval (6–7 s) and the site of attention.

We delivered brief infrared laser stimuli of 1 ms duration and a beam diameter of 5 mm to the dorsum of the left ring and index finger using a Thulium YAG laser (wavelength 2 μm, StarMedTec, Starnberg, Germany). Prior to the experiment, participants were familiarized with the use of a pain rating scale ranging from zero (no sensation) to 100 (maximal pain). On this rating scale, a value of zero indicates no sensation at all and 30 indicates the threshold for a pain sensation. Sensation higher than zero and below 30 indicates non-painful warm, rarely tactile sensations, whereas sensation at pain threshold indicates the beginning of a painful hot and stinging pain. Individual pain threshold was tested by calculating the average intensity at which subjects reported first a rating value above 30 in three ascending stimulus series and, more- over, first a rating value below 30 in three descending series of laser stimuli using successive intensity increments of 20 mJ. During the experiment subjects were comfortably seated in an electrically shielded and sound-attenuated recording chamber with their eyes closed. The experiment consisted of eight blocks in total, comprising two blocks of 20, 50, 60 and 30 stimuli, respectively. The blocks were presented in counterbalanced order (see Figure 1). High-intensity stimuli (2-fold pain threshold) and low-intensity stimuli (1.5-fold pain threshold) were delivered during all blocks. The inter-stimulus interval varied between 6 or 7 s. Before each block, subjects were instructed to attend to the stimuli at one finger. The site of stimulation was randomized, providing that no more than two successive stimuli were delivered to the same finger. Three seconds after the laser stimulus, an acoustic event (2000-Hz tone) prompted a response. The subjects’ task was to respond after stimulation of the attended finger using two keys of a response-box with their right hand to classify the intensity of the stimulus (i.e., high or low). The stimuli delivered to the other finger had to be ignored and not to be classified. To control for differences between conditions due to finger movements a third button had to be pressed. Directed attention to one finger was counterbalanced over blocks. The assistant directing the laser beam onto the different fingers was instructed via earphones about the site of stimulation. Instructions and the acoustic prompt were controlled by the Presentation software (Neurobehavioral Systems, Albany, CA, USA).

### Data Acquisition and Analysis of EEG

The EEG was recorded using 128 channels (including two EOG-channels, EASY CAP) and BrainVision Recorder software (Brain Products GmbH, Gilching, Germany) through four BrainAmp MRplus 32 channel amplifiers with a sampling frequency of 1000 Hz and a band pass filter between 0.1–250 Hz. The electrode impedance was kept below 15 kΩ. The EEG was recorded with nose reference. The data were analyzed offline using EEGLAB (Delorme and Makeig, 2004)^1^ and fieldtrip^2^, freely available open source toolboxes running under Matlab (The Mathworks, Natick, MA, USA). Firstly, data were band-pass-filtered from 0.3 to 100 Hz and downsampled to 400 Hz. Then the continuous data sets were epoched in segments from −1000 to 3000 ms. Artifact removal was done by visual inspection of all segments for the presence of artifacts such as muscular contractions. The first three trials of every block were rejected to avoid vigilance or alarming effects in the beginning of a block. For rejection of ocular and cardiac artifacts data were submitted to extended infomax independent component analysis (ICA; Bell and Sejnowski, 1995). Briefly, ICA returns a set of spatial filters, which, when matrix-multiplied with the data, yield component activations being maximally temporally independent from each other. By visual inspection of component maps and component time courses, we identified those independent components reflecting eye blinks or movements and ECG artifacts (Jung et al., 2001; Debener et al., 2005). Back-projection of the remaining non-artifactual components revealed corrected EEG data. The data were categorized for levels of intensity and attention. Noisy channels were interpolated (mean = 3.3 ± 0.8). The algorithm replaced the respective channels by the average of the surrounding clean channels, weighted by the respective distances. Given a scaling of electrode locations from −1 to 1 in all three dimensions, maximal distance for the neighboring channels to be included was 0.5. Finally data were re-referenced to common average reference.

### Spectral Analysis

Frequencies up to 40 Hz were analyzed using a sliding Hanning-window Fourier transformation with a window length of 500 ms and a step-size of 20 ms. For the analysis of frequencies higher than 40 Hz spectral analyses of the EEG data were performed using a sliding window multi-taper analysis (Mitra and Pesaran, 1999). In short, the data were multiplied by *N* > 1 orthogonal tapers and Fourier transformed, and the *N* spectral estimates are finally averaged. In case of power estimation, the spectra for each individual taper are magnitude squared after Fourier transformation. As data tapers, we used the leading 2TW-1 prolate spheroidal (slepian) sequences, where T denotes the length of the tapers and W the half bandwidth. These tapers optimally concentrate the spectral energy of the signal over the desired half-bandwidth W. Averaging across trials was finally performed in the frequency domain. A window of 300 ms length was shifted over the data with a step size of 20 ms. Spectral smoothing of 15 Hz was achieved by five slepian tapers. Time-frequency results were expressed as percent signal change relative to baseline, using a baseline interval from −1000 to −500 ms prior to stimulus onset.

### Regions of Interest

For the purpose of data reduction, several regions of interest (ROIs) were defined: a frontal ROI consisted of 26 electrodes, a central ROI around Cz of 31 electrodes, an occipital, contra- and ipsilateral ROI of 23 electrodes. Since all time frequency components were most prominent in the central ROI, this ROI was used for visualization and further statistical analysis.

### Statistical Analysis

**Figure 2:**
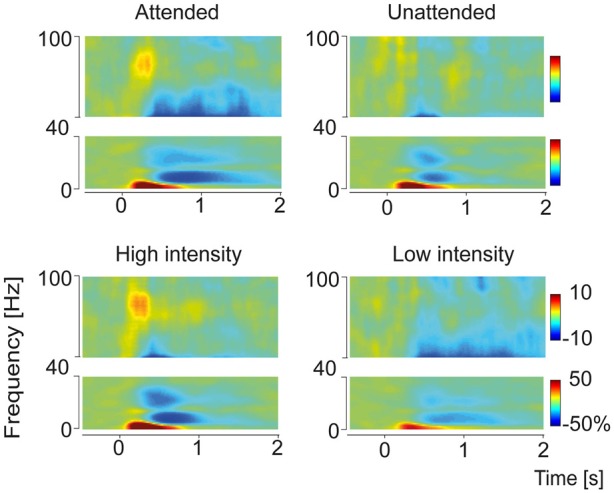
**Time-frequency representation of grand average pain-induced components sorted according to the conditions Attention (Attended and Unattended) and Intensity (High and Low).** Time-frequency representations were built from the central ROI adjacent to channel Cz. Four distinct response components could be observed: a delta-increase (4 Hz, 300 ms), an alpha decrease (10 Hz, 750 ms), a beta decrease (24 Hz, 600 ms) and a gamma increase (80 Hz, 270 ms). Power values are represented as percent change relative to the prestimulus baseline (see “Materials and Methods” Section).

For statistical analysis the statistics toolbox running under Matlab (The Mathworks, Natick, MA, USA) was used. Because the pattern of activation was most pronounced in the central region, all sensor level analyses were performed using the data of the central ROI averaged across conditions. Visually inspecting the grand average of all subjects and conditions, time-frequency windows for peak detection were chosen from the time-frequency analysis (see Figure 2). Individual power-values for the respective time-frequency-points were then fed into the source analysis. For all analyses, the critical *p*-value was set to *P* < 0.05.

### Source Localization

For each subject, data sets were averaged for the conditions intensity and attention. Linear beamforming (Van Veen et al., 1997; Gross et al., 2001; Schneider et al., 2008) was applied on the resulting average data sets to localize time-frequency components which showed significant effects in the *t*-test. A realistic 3-shell model was constructed of the Montreal Neurological Institute (MNI) template brain.^3^ Using this head model, the leadfield matrix was calculated for each grid point in a 7 × 7 × 7 mm grid. The cross-spectral density matrix between all 126 EEG-channels was calculated by means of the algorithms used for the respective frequencies. From the leadfield at the respective grid point and the cross-spectral density matrix of the respective condition, a spatial filter was constructed for each grid point, which passes activity from this location with unit gain and maximally suppresses activities from other locations. The source activities for each time-point were calculated using this common filter. The individual sources were averaged across subjects. For statistical analysis of neuronal activity a paired *t*-test was performed on the result for each grid point to estimate the signal change of each condition vs. baseline and for the difference between conditions across subjects. Subsequently *t*-values were transformed to *z*-scores. MNI-coordinates of peak voxel were transformed into Talairach-coordinates (Talairach and Tournoux, 1988) and were fed into the Talairach Daemon^4^ for classification purposes.

## Results

### Psychophysics

The classification of stimulus intensity was correct in 70 percent of the trials. This accuracy, however differed significantly between high and low stimuli (*T*_20_ = −5.4, *P* < 0.001): subjects classified only 57 percent of high-intensity trials correctly, whereas they responded correctly in 87 percent of low intensity trials. The higher incidence of correct ratings for low intensity laser stimuli was observed regardless of which finger had to be attended. There was no significant difference between the two fingers with respect to the percentage of correct intensity judgments. The percentage of errors in location, resulting from directing the attention to the wrong finger, was low and did not differ between fingers.

### Time-Frequency Analysis of Oscillatory Activity

As pain-induced oscillatory response patterns were most pronounced over the central region, statistical analyses at electrode level were performed on the central ROI. The grand average of total power across subjects and conditions revealed four distinct time-frequency response components (Figure 2): a delta power increase (maximum 4 Hz, 300 ms), an alpha power decrease (minimum 10 Hz, 750 ms), a beta power decrease (minimum 24 Hz, 600 ms) and a gamma power increase (maximum 80 Hz, 270 ms).

### Top-Down Modulation of High and Low Frequencies

**Figure 3:**
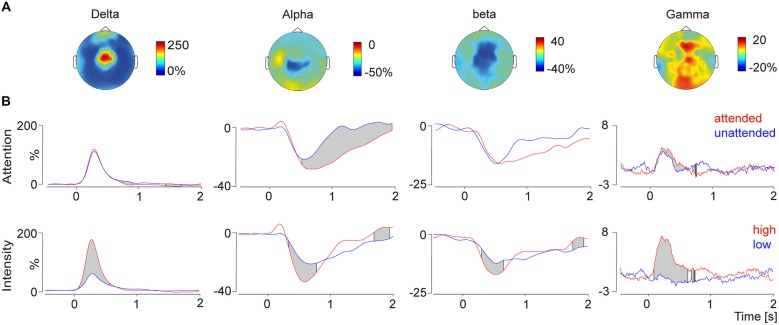
**(A)** Topographies (top view) of the delta-increase (4 Hz, 300 ms), the alpha decrease (10 Hz, 750 ms), the beta decrease (24 Hz, 600 ms) and the gamma increase (80 Hz, 270 ms). **(B)** Time course of the selected frequency bands plotted for the conditions attention (top) and intensity (bottom) modulation. The gray shades indicate significant differences between conditions calculated with a running *t*-test. Modulation of attention resulted in significant differences in the alpha and gamma band, whereas changes depending on the stimulus intensity were observed for all four time-frequency response components.

To test top-down attentional modulation pain induced peaks in the frequency domain were detected in the grand average data and the frequency bands were fed into a running *t*-test over the time domain. This analysis revealed that alpha and gamma frequencies were modulated by attention. Gamma power was higher in the attended condition compared to the unattended condition (Figure 3). Furthermore we observed a stronger alpha decrease in attended condition compared to the unattended condition.

To exclude prestimulus influence of the baseline interval on these results, the log-transformed baseline interval was fed to the running *t*-test as well. No significant differences were found between the attended and unattended conditions.

### Bottom-Up Modulation of High and Low Frequencies

Bottom-up stimulus intensity modulation of pain induced oscillations is shown in Figure 3. Significant differences between the high intensity pain stimuli and the low intensity stimuli were found in all four frequency bands. Stronger pain stimuli induced a power increase in delta and gamma power, as well as a stimulus induced power decrease in alpha and beta power. Differences of the log-transformed baseline interval showed no differences between the conditions, which was expected since the condition was not predictable.

### Source Analysis of Pain Induced Gamma Modulation

**Figure 4:**
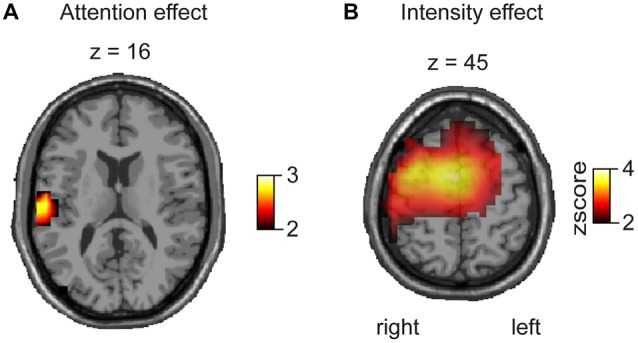
**Beamforming results represented as *z* scores, based on a voxelwise *t*-test for attentional and intensity modulation. (A)** The contrast for attentional modulation revealed a significant effect for the gamma band (80 Hz, 270 ms) in the contralateral SII/insula. **(B)** The contrast between high and low pain stimulation reveals a significant difference for the gamma band (80 Hz, 270 ms) in contralateral sensorimotor cortex and midcingulate gyrus.

In order to investigate regional specificity of the above-described gamma modulation we applied a distributed source reconstruction technique termed linear beamforming (Van Veen et al., 1997) to the data (Figure 4). Statistical maps represented by *z* scores were generated by statistical comparison between the “top-down” attention and “bottom-up” pain intensity condition (see “Materials and Methods” Section) using time-frequency peak activation cluster. Hence, the contrasts between high intensity vs. low intensity and attended vs. ignored trials were calculated in source space. Attentional bottom-up modulation (Figure 4) revealed one significant activation for gamma band activity, which was found in the contralateral insula (Talairach coordinates: *x* = 55, *y* = −24, *z* = 16). Top-down modulation of laser pain intensity revealed one significant activation located in contralateral sensory motor area and midcingulate gyrus for the gamma band (Talairach coordinates: sensory motor area: *x* = 40, *y* = −6, *z* = 45; midcingulate gyrus: *x* = 13, *y* = −5, *z* = 45).

## Discussion

Our study demonstrates effects of spatial attention and stimulus intensity on pain-induced oscillations as measured by EEG. Top-down modulation induced by directing attention to a specific finger appeared as pronounced gamma band increase and reduction of alpha band power. The attention-induced gamma-power increase was localized in the contralateral insula. Bottom-up modulation was reflected by enhancements of early delta and gamma band power and a more pronounced alpha and beta band suppression. Source localization revealed intensity-correlated gamma power increase in sensorymotor areas and the midcingulate gyrus. With our findings we could show for the first time a differentiation of pain-induced gamma band responses (GBRs) with respect to bottom-up and top-down modulation.

Recent studies reported consistently that pain stimulation elicits GBR which are linked to cortical perception and integration of pain intensity (Gross et al., 2007; Schulz et al., 2012; Zhang et al., 2012; Hauck et al., 2013) and attentional modulation of pain processing (Hauck et al., 2007). The integration of pain intensity with subjective pain perception is attributed to somatosensory areas, especially the contralateral primary SII (Gross et al., 2007; Zhang et al., 2012; Hauck et al., 2013). This assumption is consistent with our finding that bottom-up modulation, i.e., change of stimulus intensity, had a strong impact on a GBR component localized in contralateral somatosensory areas. However, it is still under debate if activations in primary somatosensory cortex (SI) exist following pain (Garcia-Larrea et al., 2003; Valeriani et al., 2004; Frot et al., 2013), because the most reported localizations following experimental laser pain in electrophysiologic experiments and intracranial recordings are the parietal operculum, SII and the cingulate cortex (CG). One of our most interesting findings is the involvement of the CG in the bottom-up modulation of the GBR. Together with SII and the insula, the CG is one of the regions most consistently activated by pain (Treede et al., 1999; Apkarian et al., 2005). However, imaging of subjects engaged in a variety of cognitive, affective and motor tasks also revealed CG activations. A multimodal integrative, rather than a specific nociceptive role of the CG is also underlined by its large receptive fields and the absence of somatotopy (Vogt, 2005). Therefore, the gamma band oscillations observed here might reflect the activity of networks involved in the multidimensional integration of pain.

In our study, pain induced GBR were also modulated by attention, consistent with an earlier MEG study of our group where we observed that top-down modulation by attention induced stronger gamma band power in bilateral sensorimotor cortex (Hauck et al., 2007). Using EEG, which is more sensitive for radial sources, we here also show an attentional modulation of GBR in the contralateral insula. The insula belongs to the limbic system and plays a major role in the integration of pain for feelings and behavior (Craig, 2002). Activity of subregions of the insula varies with pain intensity and the anterior insula represents relevance to a threat and has a role in processing stimulus novelty (Vogt and Laureys, 2005). At sensor level and in source space, attentional gamma modulation was relatively small compared to the bottom-up pain intensity effect. Potentially, this gamma component is a manifestation of the integration of the subjective pain experience. We suggest that the modulation of oscillatory response components in the gamma band may be one mechanism by which attention facilitates processing of neural pain signals relevant for bodily harm, leading to enhanced saliency of specific nociceptive input and preferential routing of the respective information through limbic and sensory pain areas. Furthermore, given the intrinsic “attention attracting” nature of pain it is possible that the difference between the attention conditions reflects differences between voluntary and automatic attention shifts. Pain, compared to other sensory stimuli attracts automatically attention towards the bodily threat. Therefore it might be possible that the unattended stimuli in our experiment were not really unattended.

Besides pain induced GBR, pain stimulation elicits power changes in other frequency bands as well (Hauck et al., 2008). Stimulus-driven bottom-up modulation induced changes in all frequency bands. The pain induced delta activity predominantly reflects slow phase-locked components that can also be observed as late laser-evoked potential (LEP) components, which are known to be enhanced by attention (Lorenz and Garcia-Larrea, 2003; Hauck et al., 2013) and by stimulus intensity. In a series of studies Legrain et al. (2002, 2003a,b) reported that the laser-evoked P2 is only enhanced by bottom-up processes, while the N2 and P3 show an enhancement for both strong as well as attended stimuli. Furthermore, a recent study by Zhang et al. (2012) should be mentioned in this context, where the authors modulated the saliency of laser pain by repetitive stimulation trains and different pain intensities. Pain induced delta power in correlation with LEP amplitudes increased with either enhanced saliency or stimulus intensity. Interestingly, no attentional effect was found on delta power. This may be due to a potential small effect of directed attention and the conjunction of slow LEP waves (N1, N2, P2, P3) in one delta power time-frequency cluster.

Consistent with other studies on pain processing (Mouraux et al., 2003; Ploner et al., 2006a,b), we observed a decrease in alpha and beta activity following pain stimulation. While the beta power decrease was modulated by stimulus intensity only, the alpha power decrease was modulated both by attention and stimulus intensity. Alpha desynchronization seems to be associated with increased mental activity and top-down processes such as attention (Klimesch, 1999; Herrmann and Knight, 2001; Neuper and Pfurtscheller, 2001; Knyazev, 2007). The alpha decrease reported here was most pronounced after strong and attended stimuli. This is consistent with the interpretation of Ploner et al. (2006a) that sensorimotor alpha band activity is a measure of the excitability of the somatosensory system. The alpha-band decrease possibly reflects the degree to which a thalamocortical “gate” is opened permitting relevant exogenous input to reach the cortex and be actively processed. The alerting function of pain may critically depend upon the ability to open relevant thalamocortical gates and inhibit task-irrelevant regions (Jensen and Mazaheri, 2010) to prepare the individual for defensive reactions. Previous studies showed that pain stimulation leads to a reduction of beta power as well (Ploner et al., 2006a). Pain induced beta power decrease has been suggested to modulate the excitability of the sensorimotor cortex and the sensitivity of pain processing and execution of adequate protective motor responses in the sensorimotor cortex modulated by emotional face expression (Senkowski et al., 2011) or multisensory stimulation (Pomper et al., 2013). Furthermore beta power may be more involved in top-down processing than in bottom-up processing and may be related to the maintenance of the current sensorimotor state (Engel and Fries, 2010). In our study, we detected a beta decrease following pain stimulation that was more pronounced after strong stimuli and showed no attentional modulation. This may be in line with the alerting role of beta power suppression in the processing of novel and relevant pain events, since in our paradigm the pain event was expected in both attentional conditions and no further attentional modulation was necessary to occur.

Recent studies in the somatosensory and visual system (Bauer et al., 2014; van Ede et al., 2014) were able to demonstrate differences in frequency responses between attentional predictability and poststimulus attentional enhancement in different frequency band. In the anticipation phase a suppression of alpha and beta oscillations occurred, whereas a stimulus bound attentional increase of gamma oscillations was present. One interpretation of these results is, that attentional modulation of alpha and beta oscillations is linked to the precision of anticipation about the stimulus, whereas gamma power is correlated to the mismatch of this expectation (Bauer et al., 2014). In our experiment, we also observed an attentional increase of gamma power, which is in line with these findings. However, due to the experimental design, the stimulus was not predictable between conditions and therefore we did not observe any anticipation or baseline differences in the alpha or beta band.

In conclusion, we were able to show different cortical oscillations and their generators to be involved in bottom-up and top-down modulation of pain processing. Most interestingly, gamma oscillations following laser-induced pain were modulated by both attention and stimulus intensity. Top-down modulation of gamma oscillation were localized in the insula, whereas bottom-up modulation of gamma oscillations were localized in sensorymotor areas and cingulate cortex. This finding suggests a key role of gamma-band oscillations in the routing of pain-related signals and the integration of nociceptive input into the multidimensional experience of pain. Future studies may address the issue of whether oscillatory response patterns of pain patients deviate from those of healthy controls. Enhanced gamma activity could be a sign of a disproportionate integration of noxious input leading to an exaggerated pain experience.

## Conflict of Interest Statement

The authors declare that the research was conducted in the absence of any commercial or financial relationships that could be construed as a potential conflict of interest.

## Footnotes

^1^www.sccn.ucsd.edu/eeglab

^2^www.ru.nl/fcdonders/fieldtrip

^3^http://www.mni.mcgill.ca

^4^http://www.talairach.org/daemon.html
